# Analgesic efficacy of oral tramadol–dipyrone combination in cats undergoing ovariohysterectomy

**DOI:** 10.3389/fvets.2026.1773463

**Published:** 2026-02-09

**Authors:** Denise Tabacchi Fantoni, Karina Velloso Braga Yazbek, Isabela Torquato de Lima, Jéssica Sperandio Cavaco, Amanda Cologneze Brito, Érica Vilela Barreto, Julia Maria Matera, Aline Magalhaes Ambrósio, Marco Aurélio Amador Pereira

**Affiliations:** 1 Department of Surgery, School of Veterinary Medicine and Animal Science, University of São Paulo, São Paulo, Brazil; 2 Technical Department, Clinical Research and Development of New Products - Biolab Sanus Farmacêutica Ltda, São Paulo, Brazil

**Keywords:** cats, metamizole, multimodal analgesia, opioid, ovariohysterectomy, postoperative care, rescue analgesia

## Abstract

**Introduction:**

Selecting an appropriate analgesic for cats can be challenging due to potential unwanted side effects, short duration of action, or unsuitable presentation for home administration. This study aimed to evaluate the analgesic efficacy and safety of a fixed-dose oral combination of dipyrone and tramadol (Sindolor Cats^®^ tablets) for postoperative pain control in cats undergoing ovariohysterectomy.

**Methods:**

Thirty-six healthy female cats were randomly assigned to three groups (*n* = 12): GTD (dipyrone 12.5 mg/kg + tramadol 2 mg/kg), GT (tramadol 2 mg/kg), and GD (dipyrone 12.5 mg/kg). Treatments were administered orally every 12 h for 5 days. Anesthesia was induced with propofol and maintained with isoflurane; all cats also received sacrococcygeal epidural lidocaine under ultrasound guidance. Pain was assessed using the CMPS-Feline and the Feline Grimace Scale (FGS). Physiological parameters (heart rate, respiratory rate) and serum cortisol were measured at baseline and at 2, 4, 6, and 24 h postoperatively. Rescue analgesia with morphine was administered when pain scores exceeded threshold values.

**Results:**

The GTD group required less rescue analgesia (1/12) compared to GT (4/12) and GD (5/12). Although not statistically significant, pain scores were consistently lower in GTD. Cortisol concentrations were significantly reduced in GTD at 4 h compared to GT and GD. Side effects such as sialorrhea were markedly lower in GTD (2/12) versus GT (9/12) and GD (12/12). No signs of cardiovascular or respiratory depression were observed in any group.

**Discussion:**

The combination of dipyrone and tramadol in a single oral formulation provided superior analgesia and fewer adverse effects than either drug alone. Cats in the combination group required less rescue medication, had significantly lower cortisol values, and experienced fewer side effects.

## 1 Introduction

Pain management is a key aspect of feline veterinary care, as pain is common in cats and can markedly affect their quality of life. Evaluating pain in cats is challenging due to their tendency to mask discomfort and display subtle behaviors. Despite these challenges, several validated pain assessment scales, such as the Glasgow Composite Pain Scale-Feline (CMPS-Feline), the Unesp-Botucatu Cat Pain Scale (UUCAPS), and the Feline Grimace Scale (FGS), have been successfully employed for pain assessments and the evaluation of analgesic efficacy of different medications (1–3).

Selecting an appropriate analgesic for cats can be challenging due to potential unwanted side effects, short duration of action, or unsuitable presentation for home administration. Opioids and non-steroidal anti-inflammatory drugs (NSAIDs) are among the most used analgesics in felines. Opioids, acting on the central nervous system’s opioid receptors to reduce pain perception, have been shown to manage pain effectively in various clinical and surgical contexts. However, side effects such as sedation, respiratory depression, constipation, and dysphoria may occur, depending on the dose and the specific drug used (4). Tramadol, an atypical opioid with a complex mechanism of action, offers a safer profile regarding cardiorespiratory depression, likely due to its milder action on opioid receptors (5). It has been utilized for managing pain related to various conditions in cats, demonstrating minimal cardiovascular and respiratory effects (4, 6, 7). However, the administration of tramadol in cats exhibits a notable variability in dosage, with recommended doses ranging from 2 to 6 mg/kg, even for ovariohysterectomy (7–9). This wide range underscores the current lack of standardized dosing guidelines in feline medicine and emphasizes the need for further research to establish optimal dosing regimens.

NSAIDs, inhibiting prostaglandin production to alleviate inflammation and pain, have also been validated for their analgesic utility in numerous painful conditions in cats, with meloxicam and robenacoxib being the two NSAIDs approved by the FDA for this species. Despite being generally safe, NSAIDs can cause gastritis, ulcers, and renal failure due to their mechanism of action (10, 11). In contrast, dipyrone, a non-opioid analgesic, is considered safe for the gastrointestinal and renal systems, with a very low risk of agranulocytosis, a concern that has been mitigated by recent meta-analyses showing no such effects in a significant number of human patients (12). Dipyrone is used in countries such as Germany, Brazil, and Poland for various pain-related conditions in humans and animals (12–15). Its use has been described in cats and dogs for pain control mainly after ovariohysterectomy (OVH) with the establishment of it effective doses in both species (8, 16, 17).

The synergistic effect of dipyrone with opioids, supports the rationale for their combination. This synergy allows lower doses of each drug, enhancing efficacy and reducing side effects. This is particularly relevant for procedures inducing mild to moderate pain, where combining analgesics has been a common practice recommended by the World Health Organization (WHO) since 1986 (18). A systematic review of the use of analgesics for OVH in dogs have shown that the association of NSAIDs with opioids decreases the percentage of animals requiring rescue medication significantly, compared to using a single type of analgesic (19). Studies in cats have obtained similar results (20, 21).

Combining analgesics in a single tablet is another common practice in human pain management with many existing associations on the market such as paracetamol and ibuprofen, ibuprofen and codeine, paracetamol and tramadol (22). For animals such associations are not available. Advantages include ease of administration and drug potentiation, potentially improving efficacy and compliance. In previous research, we demonstrated that both tramadol and dipyrone can be safely used for postoperative analgesia in female cats undergoing ovariohysterectomy, with varying percentages of rescue medication (0–40%) depending on the dose used (7, 8). This study aims to evaluate the analgesic efficacy and safety of a novel tablet formulation (Sindolor Cats®) combining dipyrone and tramadol in a single presentation. The goal is to assess whether this combination offers improved pain control and reduced adverse effects in cats undergoing elective neutering.

## 2 Materials and methods

### 2.1 Animals

A total of 36 female cats, aged between 6 months and 5 years, without breed restrictions, were selected.

Although this was a prospective clinical study involving client-owned cats, a short period of hospitalization was established to ensure safe monitoring and early detection of any delayed adverse effects. Animals were discharged on the fifth day, after final physical and laboratory evaluations. Owners provided the usual commercial diet during hospitalization, and cats were housed in individual stainless-steel cages within the veterinary hospital, with minimal environmental stress and continuous clinical supervision. This protocol was approved by the institutional ethics committee and aligned with current international welfare standards for post-operative monitoring.

Inclusion criteria included female cats from owners who agreed and authorized participation in the study (signed Informed Consent Form) and those that were easy to handle and considered clinically healthy after a clinical examination, complete blood count, and renal and hepatic biochemistry tests. Exclusion criteria were as follows: animals presenting any comorbidity (diabetes, heart disease, dermatopathies, locomotion and neurological alterations) as determined by anamnesis and clinical evaluation, and alterations in complementary exams (complete blood count, urea, creatinine, liver enzymes, total protein, and blood glucose); animals weighing less than 1.5 kg; animals with behavioral problems and difficult temperaments, which could influence the management of the study; pregnant or lactating females.

Before the surgical procedure, the animals were kept on a food fast for 8 h and a water fast for 2 h, according to the instructions given to the owner during the pre-anesthetic visit. The animals had ad libitum access to water through drinkers installed in the cages. The study is reported in accordance with the CONSORT guidelines.

### 2.2 Randomization and masking

After selection, the cats were randomly assigned by an online random sequence generator^1^ to one of three experimental groups: GTD (tramadol+dipyrone), GT (Tramadol), and GD (Dipyrone) with 12 animals each. Individuals responsible for the preparation and administration of the test medications throughout the study did not participate in the pain assessment. The tablet containing the combination of the two drugs was a new formulation (Sindolor Cats® tablets) (50 mg dipyrone/8 mg tramadol) and was administered at a dose equivalent to 2 mg/kg of tramadol + 12.5 mg/kg of dipyrone. Animals treated only with Tramadol (Cronidor® tablets) received a dose of 2 mg/kg and those with dipyrone (Dipyrone drops Biovet®) received a dose of 12.5 mg/kg. The three medications were administered orally every 12h for five consecutive days.

The first administration of the analgesics took place 1 h before the surgical procedure, and subsequent administrations were carried out every 12 h, based on the timing of the first administration. In the case of Sindolor Cats® tablets, a rounding criterion was used for the calculation of the dose for each cat in case the calculated dose did not result in a value compatible with the tablets. The quantities of tablets were rounded to the previous ¼, ensuring the provision of 1, ½, or ¼ of the tablet without the need for further fractionation. The tablets allowed for this fractionation as they were bisected, and a specific cutter was used for this purpose.

### 2.3 Anesthetic and surgical procedures

Approximately 1 h before the pre-anesthetic medication (PAM), the animals received their first treatment, according to the group they belonged to (GTD, GT, or GD), as previously described. Subsequently, the animals received acepromazine at a dose of 0.05 mg/kg (Acepran 0.2%; Vetnil Indústria Comércio Produtos Veterinários Ltda, SP, Brazil), via intramuscular (IM) route as PAM. After 20 min from the administration of the pre-anesthetic medication, venous access was established by catheterizing the cephalic vein with a 22 G catheter (Angiocath; Becton Dickinson, SP, Brazil), and the administration of Ringer’s lactate solution began at a rate of 3 mL/kg/h. Anesthetic induction was performed with intravenous propofol (5–8 mg kg^−1^; Propovan; Cristália Produtos Químicos e Farmacêuticos Ltda, SP, Brazil), titrated to effect to allow tracheal intubation. Isoflurane was used for maintenance (1 to 1.2 V%) (Isoflorane; Cristália Produtos Químicos e Farmacêuticos Ltda) in a mixture of oxygen (60%) and air, in a circular anesthesia circuit with spontaneous ventilation (Fabius Plus^R^, Drager do Brasil, Barueri, SP). Mechanical ventilation was initiated when end-tidal carbon dioxide (ETCO₂) reached approximately 40 mmHg, in order to maintain normocapnia within the physiological range for cats (35–45 mmHg). Adequate anesthetic depth was confirmed by the absence of palpebral and interdigital reflexes, lack of response to surgical stimulation, and stable cardiovascular parameters before the beginning of surgery. Sacrococcygeal epidural anesthesia was then performed using ultrasound guidance (Sonosite M-Turbo, Sonosite Inc., WA, United States) with a linear array probe (13–6 MHz HLF-38) positioned in the lumbosacral region. Proper execution of the block was confirmed by real-time observation of local anesthetic spread. Lidocaine 2% without vasoconstrictor was administered at a dose of 4 mg/kg, with the total volume adjusted to 0.36 mL/kg using 0.9% sodium chloride solution. To manage intraoperative nociception, a bolus of intravenous fentanyl (2 μg/kg) was administered if mean arterial pressure and/or heart rate increased by more than 15% compared with values obtained after anesthetic stabilization. During the surgical procedure, the following parameters were monitored: Heart rate (HR) and rhythm, peripheral oxygen saturation (SpO2), plethysmography curve, and temperature (T) with a multiparameter monitor (Triton BSM-6000 multiparametric monitor; Nihon Kohden Corporation, Tokyo, Japan). Respiratory rate (RR), concentration of inspired and expired carbon dioxide (ETCO2), concentration of inspired and expired isoflurane, through the gas analyzer (POET IQ2; Criticare Technologies Inc., WI, United States). Non-invasive blood pressure was measured using the oscillometric method with the high definition oscillometry equipment (BP Scan; Impulse, Curitiba, PR, Brazil) utilizing a cuff width of 40% of the thoracic limb circumference.

The surgical procedure was always performed by the same surgeon (JMM), a senior professor at the institution, and at the first appointment in the morning. The anesthesia team was also always the same (JSC, MAAP, DTF). Pain assessments and post-operative monitoring were carried out by the same person (ITDL) which was unaware of the analgesics administered. The date and time of the beginning and end of the anesthetic and surgical procedure, as well as the medications used, were recorded.

Blood samples were collected at baseline and before discharge to evaluate hematological and biochemical parameters, ensuring safety and tolerability of the treatment over time. The housing conditions, although standard for short-term clinical monitoring, were enriched by frequent interaction and provision of familiar food, reducing the stress of temporary hospitalization.

Heart rate and respiratory rate were recorded at baseline (BT, before the start of treatment and surgery), after clamping of the first ovarian pedicle (TP), and postoperatively on day 1 (D1) at 2, 4, 8, 10, 12, and 24 h after treatment (D1T2h–D1T24h). On subsequent days (D2–D5), HR and RR were measured at 2, 8, 12, and 24 h after the morning treatment administration. Rectal temperature (RT) and arterial blood pressure were recorded at BT, TP, and 30 min after the start of surgery (T30’).

### 2.4 Pain and sedation scoring and rescue analgesia

The animals were subjected to pain assessments using two scales: the Glasgow Composite Measure Pain Scale for Felines (CMPS-Feline) and the Feline Grimace Scale (FGS). Sedation was assessed using a 0–3 scale (0 = no effect, 1 = mild, 2 = moderate, 3 = severe), adapted from Valverde et al. (23). Analgesic intervention was instituted when scores were ≥ 5 on the CMPS-Feline and/or ≥4 on the FGS. In practice, both scales were applied simultaneously, but the decision for rescue was based on the first scale to exceed the predefined threshold. Rescue consisted of morphine (0.1 mg/kg IM), repeated once after 30 min if analgesia remained unsatisfactory. All rescue episodes were recorded, but post-rescue data were excluded from group comparisons. Animals requiring analgesic rescue received morphine at a dose of 0.1 mg/kg via the intramuscular route by an individual unaware of the experimental group. If analgesia was still unsatisfactory after 30 min, another 0.1 mg/kg of morphine was administered. All necessary rescue applications were accounted for and used in the statistical analysis to evaluate the differences between groups. However, the data for physiological variables, pain scores, and sedation scores recorded after the administration of rescue analgesia were excluded from the analysis.

For the evaluation of serum cortisol and blood glucose, blood samples were collected at BT, TP, and at 4 and 12 h after the first treatment (D1T4h and D1T12h). Samples were placed in serum tubes with separating gel, centrifuged at 1,500 g for 15 min, and the serum stored at −20 °C until analysis. Cortisol and glucose concentrations were determined at a commercial clinical pathology laboratory (Provet, São Paulo, Brazil). The complete blood count, blood biochemistry, and blood glucose tests were carried out in the Clinical Pathology Laboratory of the Department of Medical Clinic at FMVZ. Immediately before the discharge of the cats, a complete blood count and serum biochemistry were performed to monitor for potential blood dyscrasias or other laboratory alterations.

Side effects such as sialorrhea, mydriasis, behavior alteration, or any other change observed during the observation period was considered. Appetite was also evaluated.

### 2.5 Statistical analysis

The statistical analysis was conducted under the supervision of an experienced statistician. The data were analyzed using R software version 4.2.1.

After adjusting the models, the normality of the residuals was checked using the Shapiro–Wilk and Lilliefors normality tests. Homoscedasticity was verified using the Bartlett test. When these assumptions were not met (*p* < 0.05), the Box-Cox optimal power transformation was applied to the original data, and the results of the analysis of variance were evaluated again. The Tukey test was applied for comparisons of group means at each evaluation moment and for comparisons of moments within each group.

#### 2.5.1 Quantitative data

Quantitative data were analyzed using a model of analysis of variance (ANOVA) for the following time points: D0 Basal, D0 before surgery, D0 after surgery, D1-TB, D1-T14h, D1-T6h, D1-T12h, D2-T24h, D2-T12h, D3-T48h, D3-T12h, D4-T72h, D4-T12h, D5-T96h, and D5-T12h. The normality of the residuals was assessed using the Shapiro–Wilk and Lilliefors tests, and homoscedasticity was evaluated using Bartlett’s test. When the assumptions of normality and homoscedasticity were not met, the Box-Cox transformation was applied. Tukey’s test was used for multiple comparisons of means between groups and time points.

#### 2.5.2 Categorical data

For categorical data, Fisher’s exact test was used, with corrections applied to maintain a significance level of 5% when necessary. A *p* value < 0.05 was considered significant.

## 3 Results

**Figure 1:**
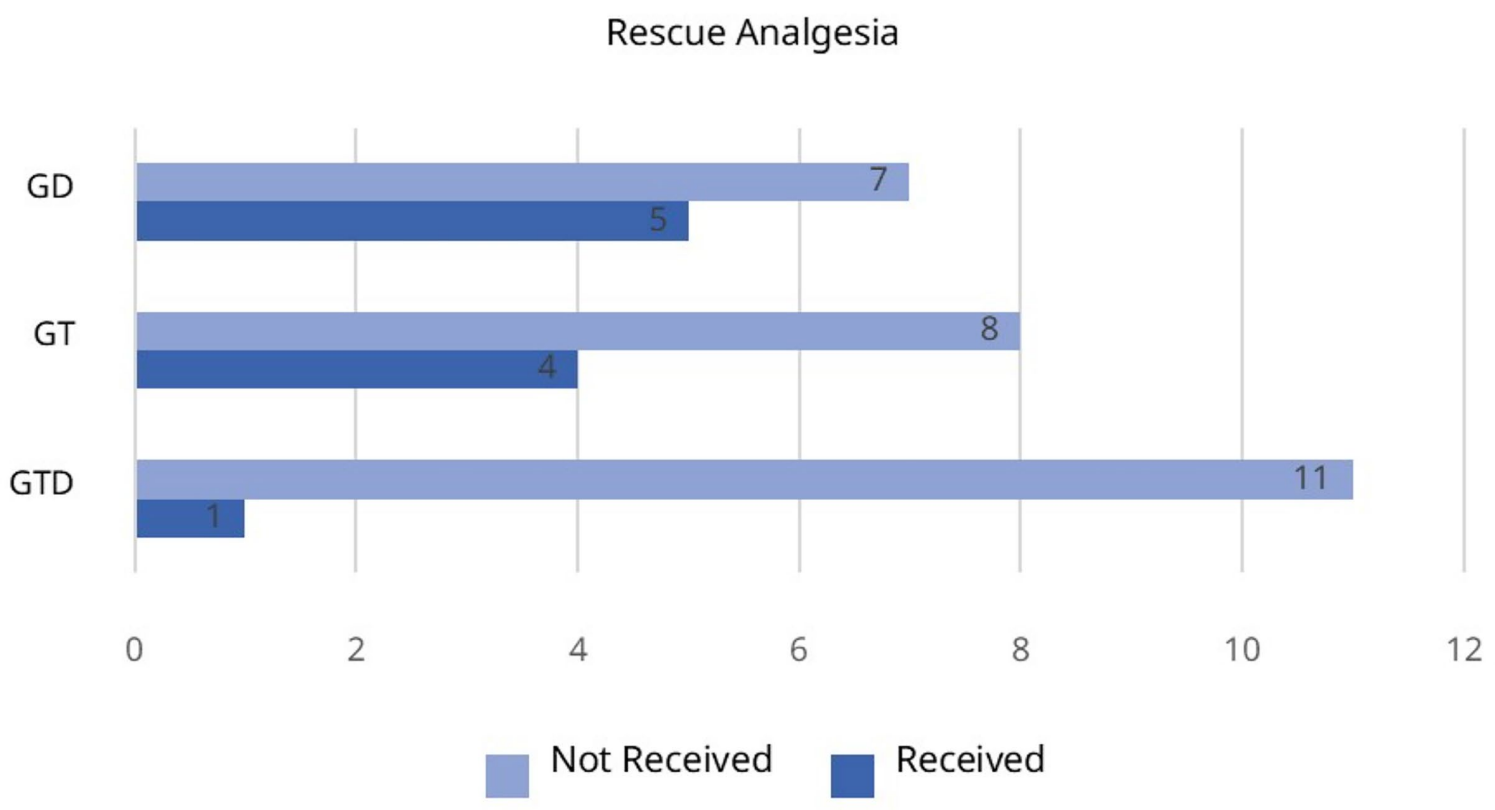
CONSORT flow diagram illustrating randomization, group allocation, and data analysis for cats receiving tramadol and dipyrone (GTD group), tramadol (GT), or dipyrone (GD) prior to ovariohysterectomy.

A total of 43 cats were evaluated for this study, with 36 included after excluding seven due to challenging behavior during the screening and one due to altered blood sample (Figure 1). The procedures took place in the morning, with the surgeries lasting 33 ± 2, 32 ± 3 and 37 ± 2 min for GTD, GT and GD, respectively, with no differences among them. Furthermore, there were not significant differences in weight and age among the groups. None of the cats required rescue analgesia during surgery.

**Figure 2:**
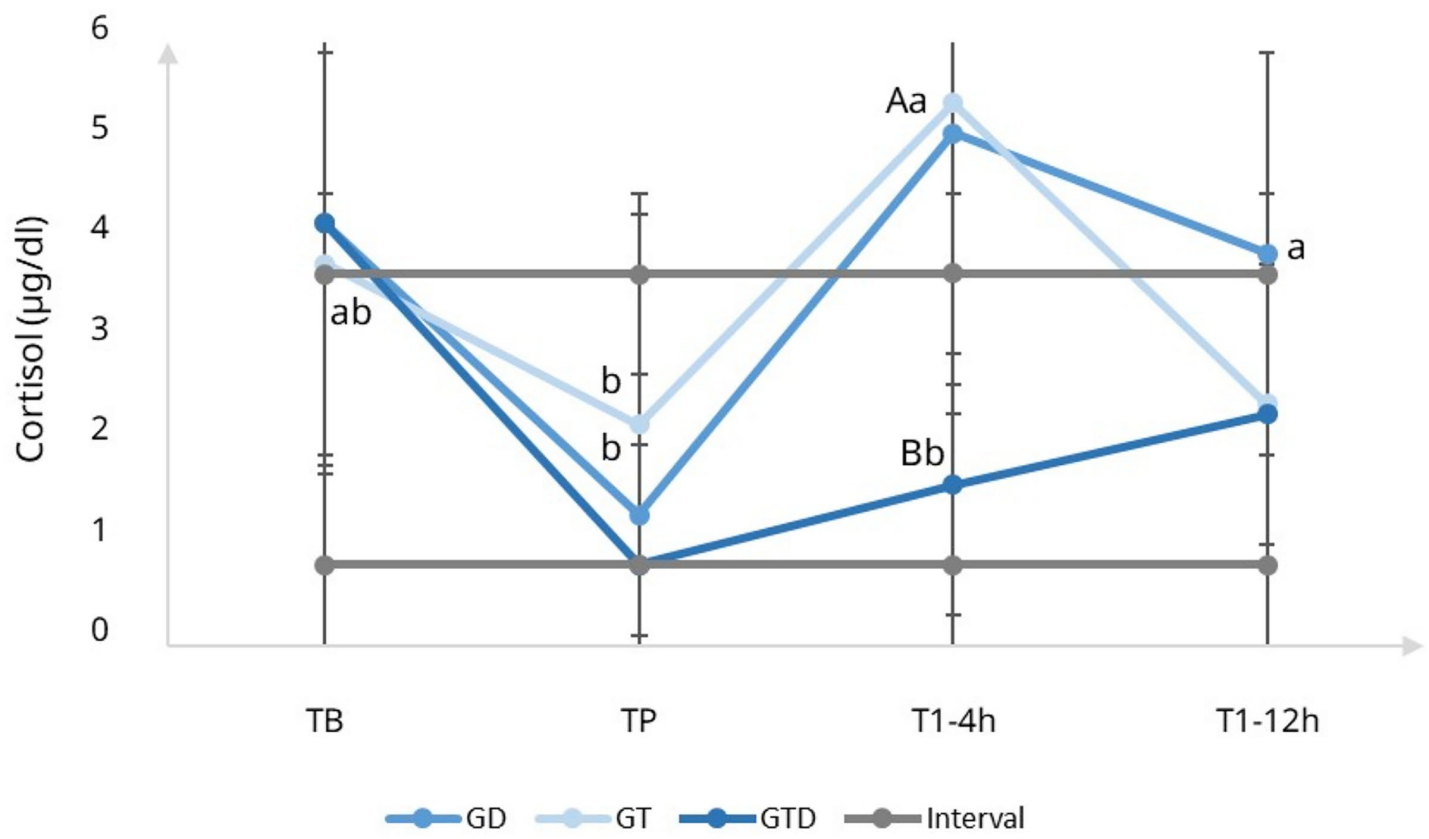
Incidence of rescue analgesia in cats undergoing ovariohysterectomy and treated with tramadol (GT), dipyrone (GD), or the combination (GTD). Only 1/12 cats in GTD required rescue compared with 4/12 in GT and 5/12 in GD. Although not statistically significant (*p* > 0.05), the combination showed the lowest rescue rate.

Rescue analgesia within 0–24 h occurred in 1/12 cats in GTD (8.3%), 4/12 in GT (33.3%), and 5/12 in GD (41.7%). Compared with GTD, the odds of requiring rescue were higher in GT (OR 5.5, Fisher’s *p* = 0.317; RR 4.0, 95% CI 0.52–30.8) and in GD (OR 7.9, Fisher’s *p* = 0.155; RR 5.0, 95% CI 0.68–36.7) (Figure 2). Although not statistically significant, these effect sizes suggest a clinically relevant reduction in rescue analgesia in the GTD group.

According to the CMPS-Feline, significant differences (*p* < 0.05) were observed at baseline (BT), where GTD differed from GD but not from GT. GD did not differ significantly from GT. At T3–T48h, GTD again differed from GD, but not from GT. Within each treatment group, a significant time effect was observed (*p* < 0.05), with mean pain scores progressively decreasing from T1–T4h until the end of the observation period. Since no further statistical differences were detected among treatment groups after day 1, the data presented focus on the values obtained at 12 and 24 h after treatment. The FGS showed similar results, with pain scores decreasing during the first hours and fewer animals requiring rescue medication (Table 1). No rescue analgesia was required beyond the first 24 h in any of the treatment groups. Sedation scores did not differ among the groups.

**Table 1:** Incidence of cats requiring rescue analgesia during the first 12 h after treatment, assessed with the CMPS-Feline and the Feline Grimace Scale (FGS).

Pain scales										
CMPS-Feline						FGS				
Groups	D1T4h	D1T6h	D1T8h	D1T10h	D1T12h	D1T4h	D1T6h	D1T8h	D1T10h	D1T12h
GTD	1 (0–1)	0 (0–0)	0 (0–0)	0 (0–0)	0 (0–0)	0 (0–0)	0 (0–0)	0 (0–0)	0 (0–0)	0 (0–0)
GT	0 (0–0)	2 (0–2)	0 (0–0)	1 (0–1)	0 (0–0)	0 (0–0)	2 (0–2)	0 (0–0)	0 (0–0)	0 (0–0)
GD	0 (0–0)	3 (0–3)	1 (0–1)	0 (0–0)	0 (0–0)	0 (0–0)	0 (0–0)	0 (0–0)	0 (0–0)	0 (0–0)

Time points are shown as D1T4h (4 h after treatment on day 1), D1T6h (6 h), D1T8h (8 h), D1T10h (10 h), and D1T12h (12 h).

**Figure 3:**
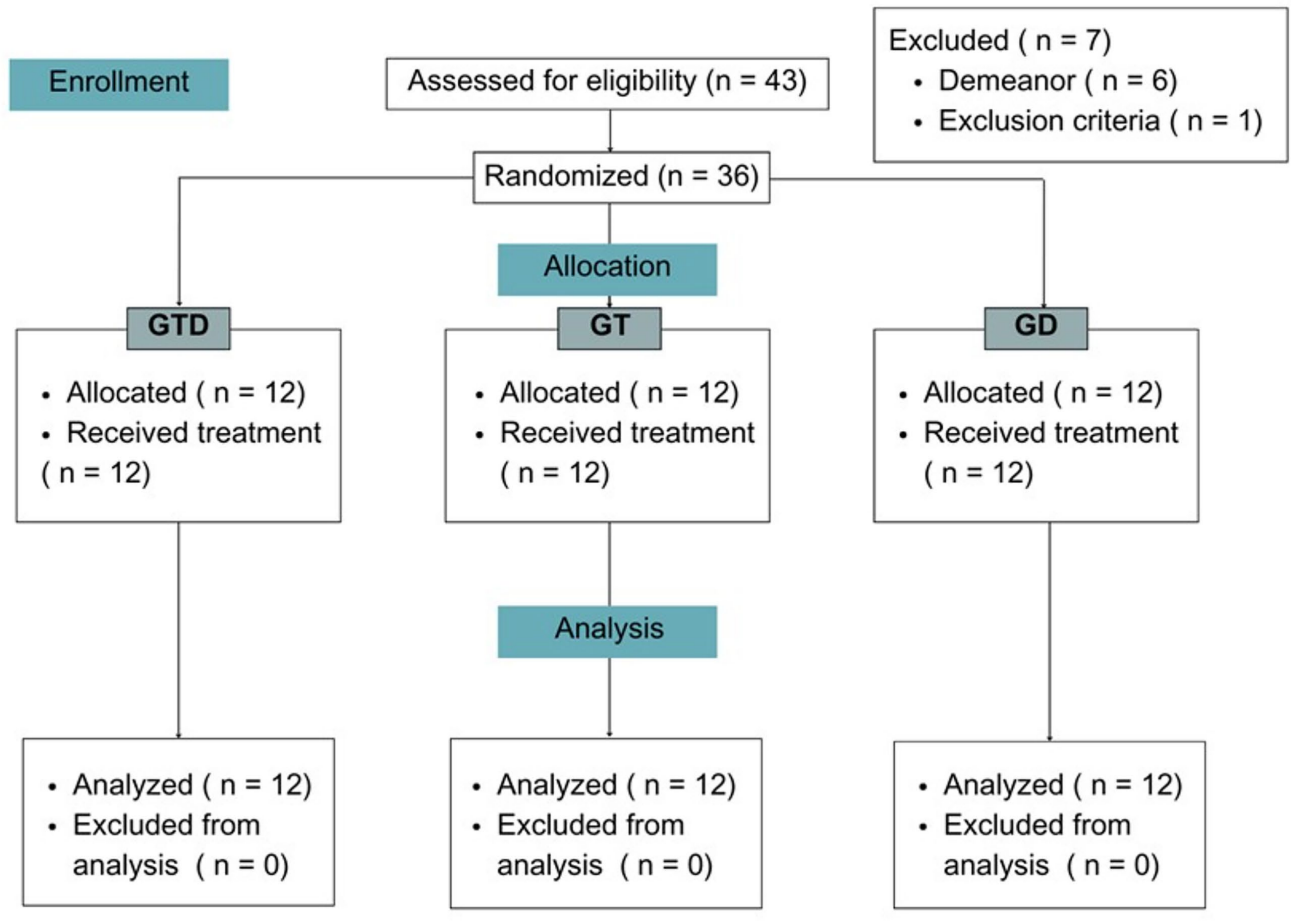
Cortisol values (μg/dl) expressed as mean ± SD in the GD, GT, and GTD groups at baseline (TB), time after clamping of the 1st ovarian pedicle (TP), and 4 and 12 h after the first treatment (T1-4 h and T1-12 h, respectively). There was a difference between the groups within each time (*p* < 0.05) (different letters). There was a difference in the time effect within each group (*p* < 0.05). Means followed by capital letter and lowercase letter in the column, respectively, do not differ by the Tukey test (*p* > 0.05). Cortisol reference interval (0.8–3.7 μg/dL).

All groups showed slightly elevated cortisol levels at baseline, likely due to the stress associated with the first handling (Figure 3). The GTD group had significantly lower cortisol levels compared to the GT and GD groups at DT1 (4 h). At T12, only the dipyrone group differed significantly from both GTD and GT. No differences of glucose levels were observed during the evaluation period.

Regarding HR, there was a significant difference between the groups within D3-T48h (*p* < 0.05), where the GTD group showed a lower heart rate (HR 186.7 beats per minute) compared to the GD (HR 220.0 bpm). No other difference was verified among the three groups during the study. Analysing the values of fR, at the timepoint D1-T6h, the GTD group showed the lowest respiratory rate followed by the GT group and GD group fR (33.3 ± 6.6, 37.7 ± 7.3, and 45 ± 12, respectively). Other differences were noted between the GTD and GT at D2-12hs, GD and GT, and GD and DTD at D2-24hs and finally, GD and GT at D3-24hs, with values reaming in the normal range for the specie.

No clinically relevant hematological or biochemical abnormalities were observed. The full dataset of hematological and biochemical values is provided as supplementary material.

The most frequent side effects observed were sialorrhea and mydriasis. Sialorrhea occurred in all cats of GD, 9 cats of GT and 2 cats of GTD. Mydriasis occurred in 9 cats of the GD, 10 animals of the GTD and all animals from GT. No other side effect was verified during the evaluation period. Noteworthy was the number of animals showing mydriasis in the tramadol group which decreased along the observation period (Table 2).

**Table 2:** Number of animals showing side effects as sialorrhea and mydriasis according to administration day and treatment group (GTD, GT, GD).

Side effects	Sialorrhea					Mydriasis				
Groups	D1	D2	D3	D4	D5	D1	D2	D3	D4	D5
GTD	2	2	2	2	2	9	9	9	9	9
GT	6	6	4	1	2	11	11	11	11	10
GD	11	11	11	11	10	9	9	4	5	5

## 4 Discussion

To our knowledge, this is the first study to evaluate the combination of dipyrone and tramadol formulated in a single tablet for cats. The results demonstrate that it provides better pain relief in female cats undergoing ovariohysterectomy than either drug alone. Cats in the combination group required less rescue medication, had significantly lower cortisol values, and experienced fewer side effects.

Various guidelines from different associations recommend multimodal analgesia, involving multiple analgesic drugs with different mechanisms of action, to provide effective pain relief in humans and various species, including dogs and cats (11, 24, 25). Combining opioids with paracetamol or dipyrone for pain relief in humans is a common practice worldwide. In this context, the FDA has approved several combination analgesics, such as paracetamol with codeine (e.g., Tylenol® with Codeine) and paracetamol with tramadol (e.g., Ultracet®). In Brazil, besides these medications, other combinations containing dipyrone rather than paracetamol are approved for humans. These combinations are intended to enhance pain relief by utilizing the synergistic effects of the individual components (26, 27).

Our study explores a similar strategy, offering a novel option for feline pain management. Unlike existing combinations, the dipyrone–tramadol single-tablet formulation may simplify dosing, improve compliance, and enhance outcomes. Additional benefits include cost-effectiveness and reduced risk of medication errors.

Several studies have shown benefits of this combination, with or without NSAIDs, in managing pain from osteoarthritis in cats (21), post-mandibulectomy in dogs (28), and chronic cancer pain in dogs (15). The complementary pharmacologic profiles of tramadol and dipyrone support their combination. Tramadol and its active metabolite M1 are *μ*-opioid receptor agonists that inhibit noradrenaline and serotonin reuptake, providing analgesia without the cardiorespiratory depression seen with full μ-agonists (5). Dipyrone, first introduced in 1922, has a complex mechanism of action. It primarily inhibits cyclooxygenase (COX), reducing prostaglandin production (8). Additionally, dipyrone modulates pain pathways in the central nervous system and enhances the release of endogenous opioids. Recent studies suggest that dipyrone may interact with the endocannabinoid system, specifically involving cannabinoid receptors, contributing to its analgesic properties (29). This dual action on both peripheral and central mechanisms, along with its interaction with cannabinoid receptors, makes dipyrone an effective analgesic. It is widely used in both human and veterinary medicine across many countries (30–32). A recent study involving 384,668 Brazilian patients found that dipyrone, alone or in combination, was used in 88% of cases requiring analgesia (30). This widespread use is attributed to its safer profile compared to NSAIDs and its synergistic action with other drugs.

No published study has evaluated the pharmacokinetics of dipyrone–tramadol combination in cats.

A study using 25 mg/kg of dipyrone in cats showed prolonged elimination half-life of the active metabolite 4-aminoantipyrine (AA): 27.50 ± 11.21 h (IV) and 15.61 ± 8.69 h (IM). The authors suggest this difference may result from the lack of a specific acetylating enzyme in cats. This pharmacokinetic aspect, along with our previous clinical findings, supported the choice of a 12.5 mg/kg BID dosing regimen.

In this study, the lower dose of dipyrone combined with tramadol provided effective analgesia, with only one cat requiring rescue at a single time point during the 5-day follow-up. In contrast, the groups receiving tramadol or dipyrone alone had 4 and 5 animals needing rescue analgesia, respectively. These findings agree with previous studies reporting 30–40% rescue rates when dipyrone at 25 mg/kg was used alone for neutering female cats or dogs (8, 16, 33). In a previous study by our group in cats, we verified that with the 25 mg/kg dose daily, the percentage of cats needing rescue medication doubled compared to those receiving 12.5 mg/kg every 12.5 h, being 40 and 20%, respectively (8). Considering the low tramadol dose (2 mg/kg), the synergistic effect of the combination is implied. Brondani et al. (20) reported a 50% need for rescue medication with this dose in cats submitted to OVH, a result very similar to our prior findings (7). Metamizole and tramadol undergo rapid hepatic metabolism to their active compounds, 4-aminoantipyrine and O-desmethyltramadol (M1), respectively. Potential pharmacokinetic or pharmacodynamic interactions between these metabolites cannot be excluded, particularly considering that both contribute to central and peripheral analgesic mechanisms. Experimental evidence has previously suggested a synergistic analgesic interaction between tramadol and metamizole in dogs (15). In the present study, however, no additive or unexpected adverse effects were observed, supporting the clinical tolerability of this combination in cats.

To date, no pharmacokinetic study has simultaneously quantified tramadol, O-desmethyltramadol, and dipyrone metabolites following co-administration in cats. Nevertheless, the individual pharmacokinetics of tramadol and M1 in this species (6, 34), as well as those of the two major active dipyrone metabolites, 4-methylaminoantipyrine (MAA) and 4-aminoantipyrine (AA) (35, 36), are well characterized. Importantly, cats produce the M1 metabolite more efficiently than dogs, resulting in a more pronounced opioid-mediated effect of tramadol, which may have contributed to the observed analgesic efficacy of the combination.

Furthermore, experimental data in donkeys have shown that metamizole co-administration increases tramadol and M1 bioavailability in a dose-dependent manner, likely through competition at cytochrome P450 enzymes (37). Although extrapolation across species should be made with caution, these findings provide biological plausibility for metabolic interactions that may enhance analgesic efficacy when both drugs are administered concomitantly. In the present study, the fixed-dose combination was clinically well tolerated over the five-day treatment period, with no relevant hematological or biochemical alterations observed. Nonetheless, dedicated pharmacokinetic and pharmacodynamic studies of this combination in cats are still lacking and represent an important avenue for future research.

Cortisol levels did not differ among groups intraoperatively. Epidural lidocaine provided effective antinociception in all cats. The choice of this protocol was due to the limited duration of lidocaine, which would not interfere with postoperative pain evaluation (38). Four hours post-administration, the combination group had significantly lower cortisol levels than the dipyrone and tramadol groups, suggesting superior analgesia. At 12 h, the dipyrone group showed higher levels than the others.

Although longer than in some feline analgesia studies, the 5-day monitoring period was essential to detect delayed or cumulative adverse effects, such as hematologic or gastrointestinal changes after repeated dosing. Furthermore, all cats were owned and returned to their homes after recovery. This temporary hospitalization was agreed upon with informed consent and allowed close clinical surveillance, ensuring ethical and safe conduct throughout the study.

Although opioids may cause respiratory or cardiovascular depression in cats, no such events were observed in any group. This is consistent with previous studies that have evaluated the use of tramadol in cats (39–41). Another potential adverse effect of analgesic drugs in cats is disorientation or dysphoria. In our study, we observed minimal disorientation or dysphoria in the cats receiving the combination of dipyrone and tramadol.

Salivation was the most frequently observed adverse event and is a well-documented response in cats following oral administration of bitter-tasting drugs, including analgesics. This reaction is generally considered non–clinically relevant when transient and often reflects a sensory or taste-related response rather than systemic intolerance. Steagall et al. have reported that tramadol’s bitter taste contributes to salivation and nausea in cats, which may negatively impact treatment compliance and has limited its clinical acceptance. In the present study, salivation was observed in a smaller proportion of cats receiving the dipyrone–tramadol combination, with mean proportions of 16.6% in the combination group, 33.3% in the tramadol group, and 83% in the dipyrone group over the five-day treatment period.

From a practical standpoint, achieving comparable multimodal analgesic coverage with isolated dipyrone and tramadol would require separate administrations, potentially increasing handling time, oral exposure, and the likelihood of palatability-related aversive responses. The use of a single tablet containing both active ingredients may therefore represent a practical advantage in feline postoperative care, where treatment adherence is a recognized challenge. From a clinical perspective, transient salivation is commonly managed by caregivers and veterinarians and is often accepted as a trade-off when effective analgesia is achieved. Taken together, these findings suggest that the dipyrone–tramadol combination provides an appropriate balance between analgesic efficacy and practical administration, and that palatability-related salivation should be interpreted as an expected and manageable characteristic rather than a limiting factor for clinical use.

The 5-day evaluation period allowed observation of cumulative dosing effects and delayed adverse reactions. Adverse effects may vary by individual and protocol. Monteiro et al. (39) reported that 6.25% of cats receiving 3 mg/kg BID tramadol for osteoarthritis showed adverse effects (vomiting, sedation, mydriasis), and 18.75% had mild euphoria. Brondani et al. (20) found no differences in food intake after 3 days of tramadol vs. vedaprofen or the combination, and no other adverse effects were reported. Therefore, it is important for veterinarians to monitor cats closely for adverse effects and adjust the analgesic protocol as needed.

No blood dyscrasias were observed after 5 days of treatment. Previous studies in dogs and cats also report no such effects (8, 33). In a study that specifically investigated hematologic changes, no abnormalities were found (40). In contrast, when combined with tramadol or NSAIDs for long-term pain control in dogs with cancer, it provided excellent analgesia without adverse effects (15).

A limitation of the present study is the absence of an *a priori* statistical power analysis. Although clinically relevant differences were observed between groups, particularly regarding the proportion of animals requiring rescue analgesia, the relatively small sample size may have limited the ability to detect statistically significant differences for this outcome. Therefore, the results related to rescue analgesia should be interpreted with caution, and larger prospective studies are warranted to confirm these findings.

## 5 Conclusion

The dipyrone–tramadol combination (Sindolor Cats®) provides effective analgesia with minimal adverse effects in female cats undergoing ovariohysterectomy. Multimodal analgesia with this combination should be considered in feline surgical patients to ensure adequate pain control and welfare. Monitoring for adverse effects and protocol adjustment remains essential.

## Data Availability

The original contributions presented in the study are included in the article/supplementary material, further inquiries can be directed to the corresponding author.

## Footnotes

1
http://www.randomization.org
